# Supplementary material for: EEG-based brain-computer interface enables real-time robotic hand control at individual finger level
Source: Nat Commun. 2025 Jun 30;16:5401. doi: 10.1038/s41467-025-61064-x (PMC12209421; doi:10.1038/s41467-025-61064-x)
Supplement: Supplementary file 1 — Supplementary Information [file 41467_2025_61064_MOESM1_ESM.pdf]

Supplementary Materials

of

EEG-based Sensorimotor Rhythm Brain-Computer Interface Enables Real-time Robotic Hand Control at Individual Finger Level

Yidan Ding[1], Chalisa Udompanyawit[2], Yisha Zhang[1], and Bin He[1,2,3,*]

[1] Department of Biomedical Engineering, Carnegie Mellon University

[2] Department of Electrical and Computer Engineering, Carnegie Mellon University

[3] Neuroscience Institute, Carnegie Mellon University

* Correspondence: bhe1@andrew.cmu.edu

List of Supplementary Materials

Supplementary Fig. S1. MI online performance.

Supplementary Fig. S2. Example feature maps for deep learning online MI decoding.

Supplementary Fig. S3. The effect of EEG input from the sensorimotor region on offline finger ME and MI decoding performance.

Supplementary Fig. S4. Effect of EEG input from distinct cortical subregions on offline decoding performance for finger ME and MI.

Supplementary Fig. S5. ME online performance.

Supplementary Fig. S6. Example feature maps for deep learning online ME decoding.

Supplementary Fig. S7. Machine and human learning effects for ME-based robotic control tasks.

Supplementary Fig. S8. Simulated online decoding using deepEEGNet for MI-based robotic control tasks.

Supplementary Fig. S9. Offline FBCSP decoding results on MI robotic finger control data over 5 training sessions.

Supplementary Fig. S10. Alpha ERD at channel C3 during MI robotic finger control.

Supplementary Fig. S11. Inter-finger distance for ERD activation.

Supplementary Fig. S12. Comparison of electrophysiological activations between BCI responders and non-responders.

Supplementary Fig. S13. Low-frequency EEG activity during finger ME and MI tasks.

Supplementary Fig. S14. Comparison of offline EEGNet decoding performance for ME and MI tasks using EEG signals filtered with different bandpass settings.

Supplementary Fig. S15. Group averaged decoding probability of the target class over time.

Supplementary Fig. S16. The effect of EEG input with different channel densities on offline finger ME and MI decoding performance.

Supplementary Fig. S17. Comparison of online performance between ME and MI.

Supplementary Fig. S18. Visualization of the temporal features derived from within-subject trained EEGNet-8,2 models for Subject 07 during the finger ME task, Subject 07 during the finger MI task, and Subject 08 during the finger ME task.

Supplementary Fig. S19. Online performance comparison under different feedback conditions (n = 10).

Supplementary Table S1. Model architecture for deepEEGNet.

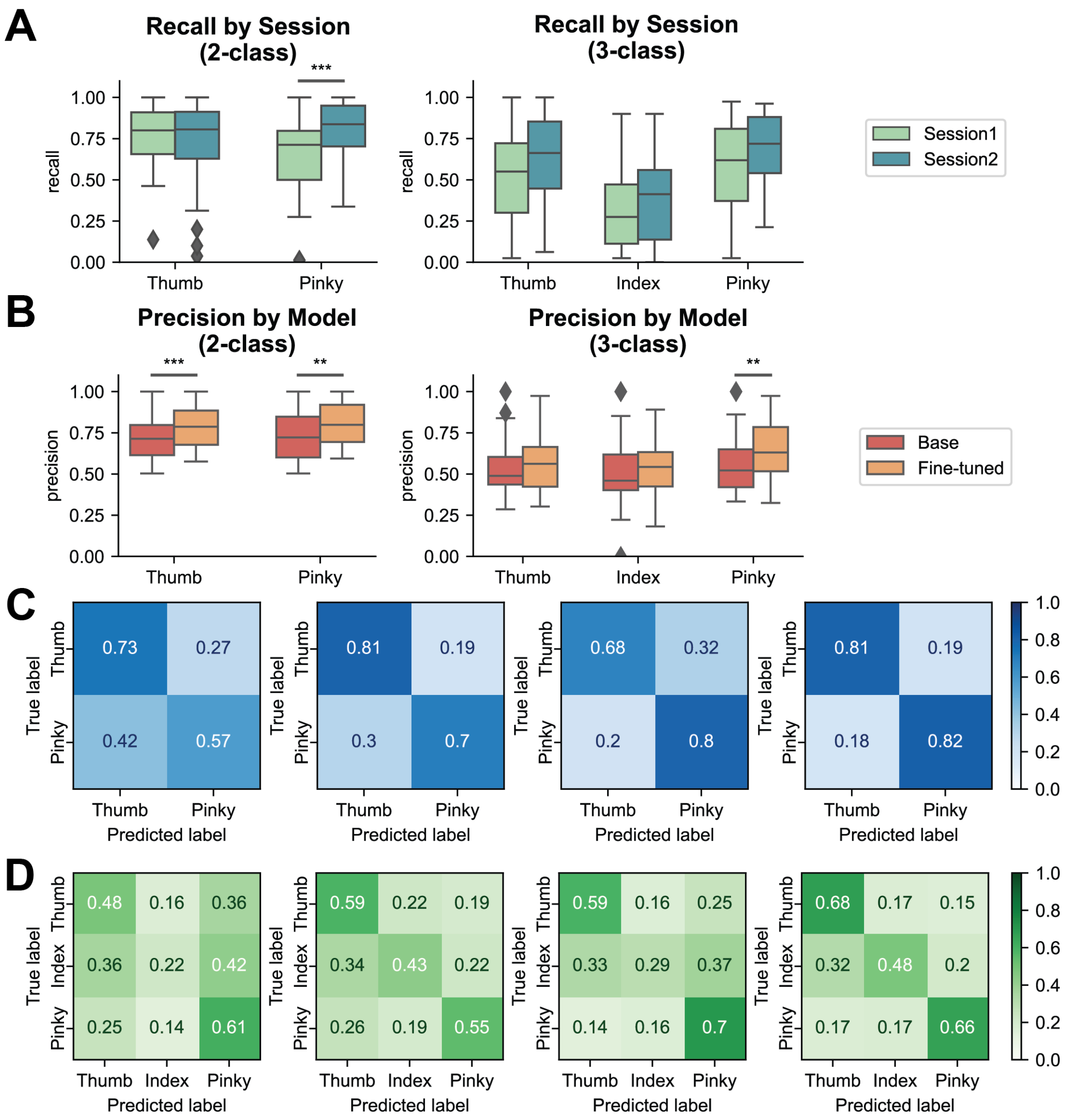

Supplementary Fig. S1. MI online performance. (**A** and **B**) Group-level recall of 2-finger and 3-finger online MI decoding results across sessions (**A**) and group-level precision of 2-finger and 3-finger MI decoding results for different classifiers (**B**) (n = 21 subjects). The center lines indicate the median value. The boxes extend from the lower quartile to the upper quartile. Diamonds indicate outliers that are more than 1.5 times the interquartile range above the third quartile or below the first quartile. Comparisons between the precision across sessions and recall across models were conducted using a two-tailed Wilcoxon signed-rank test with Bonferroni correction for multiple comparisons (*** if $p < 0.001$, * if $p < 0.05$). P-values: 1.23e-05 (A, Pinky, 2-class), 0.0006 (B, Thumb, 2-class), 0.0032 (B, Pinky, 2-class), 0.0012 (B, Pinky, 3-class). (**C** and **D**) Confusion matrixes for 2-finger (**C**) and 3-finger (**D**) online MI decoding results across sessions and different model types, which quantifies the performance of a classifier by class. The values represent the proportion of predictions for each true label classified as each predicted label. From left to right are Session 1 with the base model, Session 1 with the fine-tuned model, Session 2 with the base model, and Session 2 with the fine-tuned model, respectively.

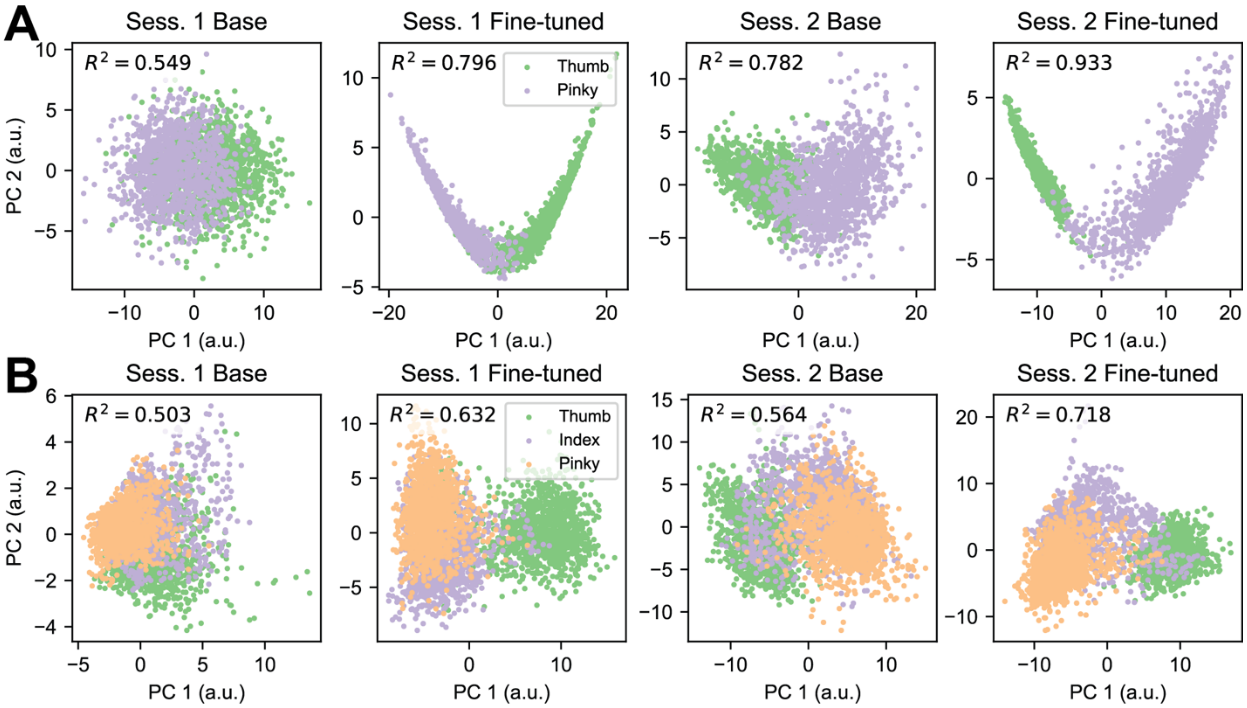

Supplementary Fig. S2. Example feature maps for deep learning online MI decoding. (A) Distribution of the first two principal components from Principal Component Analysis (PCA) of intermediate-layer features extracted from the flatten layer outputs of the deep learning decoder for 2-class MI tasks. The R-squared score from linear regression between the intermediate-layer features and the task labels was annotated in the figure. **(B)** Distribution of the first two principal components from PCA of intermediate-layer features extracted from the flattened layer outputs for 3-class MI tasks. Class discriminability was enhanced through fine-tuning and the increased training size in both 2-class and 3-class scenarios.

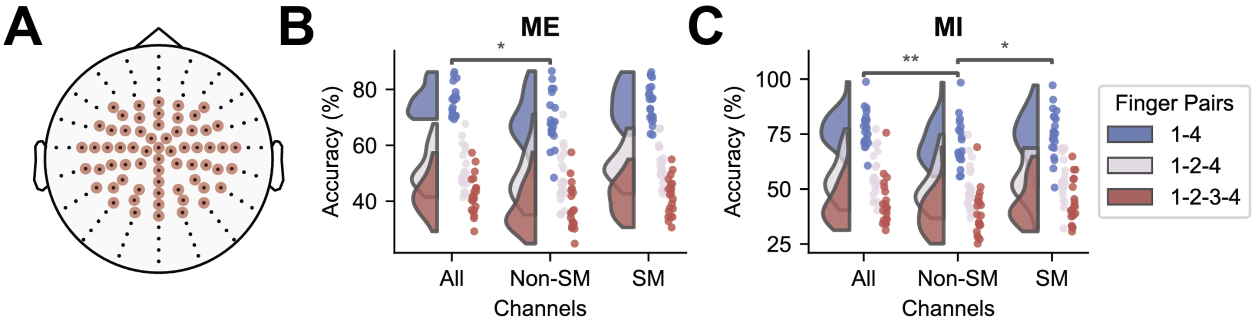

Supplementary Fig. S3. The effect of EEG input from the sensorimotor region on offline Finger ME and MI decoding performance. (**A**) EEG electrode layout, with channels from the sensorimotor region highlighted in red. (**B** and **C**) Offline decoding results for finger ME (**B**) and MI (**C**) tasks (n = 21 subjects). Decoding performance was compared using different EEG inputs. "All" refers to the condition where all 128 channels were used for decoding, "Non-SM" refers to the condition where only channels outside the sensorimotor region indicated in (A) were used for decoding, and "SM" refers to the condition where only channels inside the sensorimotor region were used for decoding. Statistical analysis was conducted using a two-way repeated-measures ANOVA with the main effects of Finger Pairs (1-4, 1-2-4, and 1-2-3-4) and Channels (All, Non-SM, and SM). The ANOVA identified a significant main effect of Channels for ME ($F = 5.785$, $p = 0.006$) and MI ($F = 5.785$, $p = 0.\ 006$) tasks. A post-hoc pairwise comparison was then performed on Channels using FDR-corrected two-tailed Wilcoxon signed-rank test results (** if $p < 0.01$, * if $p < 0.05$). The 128-channel input significantly outperformed the non-sensorimotor region. In MI decoding, using the sensorimotor region as input yielded significantly better performance than using only the non-sensorimotor region. P-values: 0.0188 (B, All vs. Non-SM), 0.0041 (C, All vs. Non-SM), 0.0394 (C, Non-SM vs. SM).

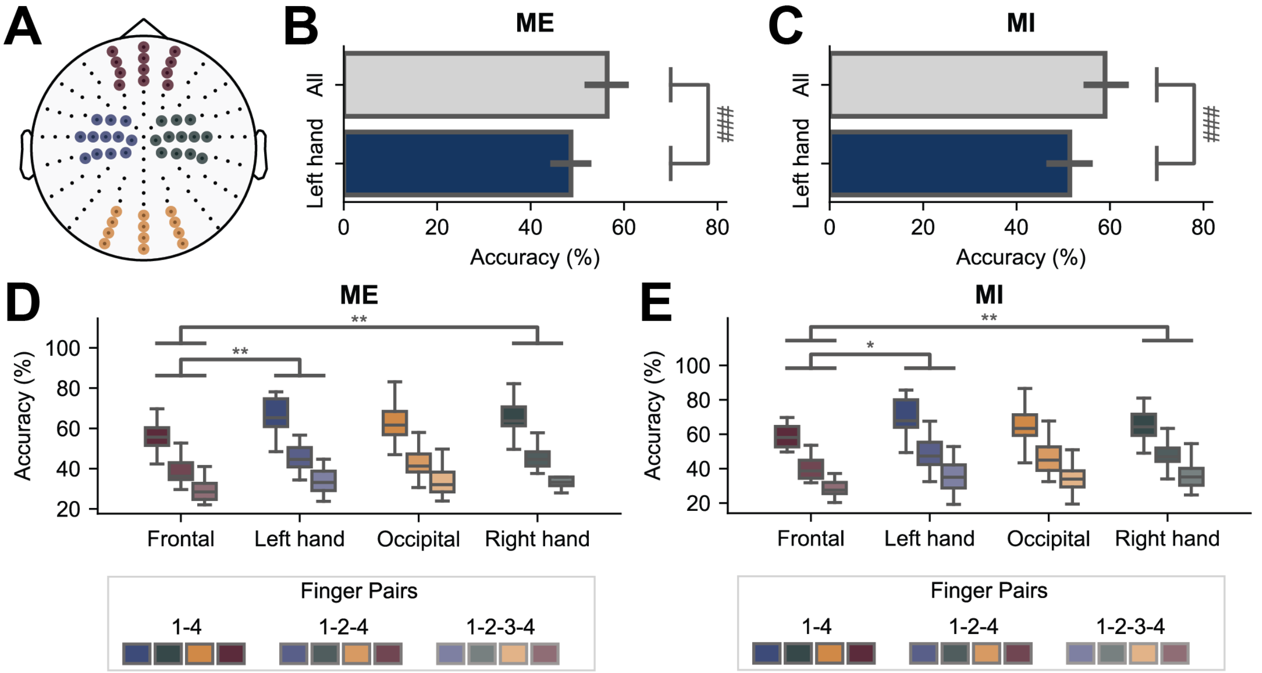

Supplementary Fig. S4. Effect of EEG input from distinct cortical subregions on offline decoding performance for Finger ME and MI. (**A**) EEG electrode layout, with channels overlying the left hand knob (dark blue), right hand knob (dark green), occipital (yellow), and frontal (dark red) regions highlighted. (**B** and **C**) Comparison of offline decoding accuracy between whole-scalp (All) and left hand knob (Left hand) inputs for finger ME (**B**) and MI (**C**) tasks (n = 21 subjects). Error bars represent the standard error. Statistical analysis was conducted using a two-way repeated-measures ANOVA with the main effects of Finger Pairs (1-4, 1-2-4, and 1-2-3-4) and Regions (All and Left hand). Main effect of Regions: ### if $p < 0.001$. P-values: 1.79e-05 (B), 0.0005 (C). (**D** and **E**) Offline decoding accuracy for finger ME (**D**) and MI (**E**) tasks using EEG input from four subregions (n = 21 subjects). The center lines indicate the median value. The boxes extend from the lower quartile to the upper quartile. The whiskers span up to 1.5 times the interquartile range. Statistical analysis was conducted using a two-way repeated-measures ANOVA with the main effects of Finger Pairs (1-4, 1-2-4, and 1-2-3-4) and Regions (Frontal, Left hand, Right hand, and Occipital). The ANOVA identified a significant main effect of Regions for ME ($F = 5.426$, $p = 0.002$) and MI ($F = 4.044$, $p = 0.011$) tasks. A post-hoc pairwise comparison was then performed on Channels using FDR-corrected two-tailed Wilcoxon signed-rank test results (** if $p < 0.01$, * if $p < 0.05$). P-values: 0.0064 (D, Frontal vs. Left hand), 0.0017 (D, Frontal vs. Right Hand), 0.0303 (E, Frontal vs. Left hand), 0.0070 (E, Frontal vs. Right Hand).

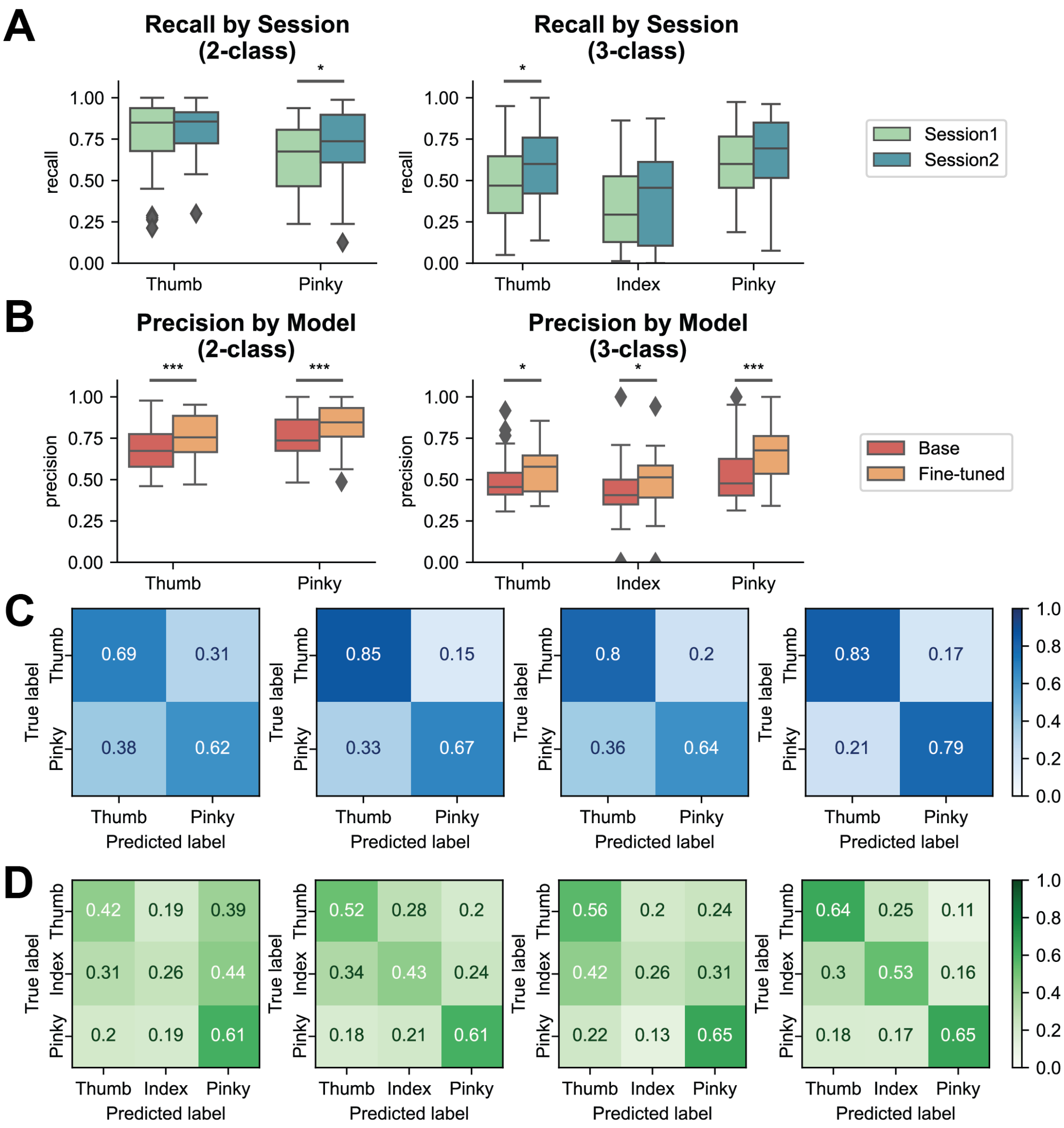

Supplementary Fig. S5. ME online performance. (**A** and **B**) Group-level recall of 2-finger and 3-finger online ME decoding results across sessions (**A**) and group-level precision of 2-finger and 3-finger ME decoding results for different classifiers (**B**) (n = 21 subjects). The center lines indicate the median value. The boxes extend from the lower quartile to the upper quartile. Diamonds indicate outliers that are more than 1.5 times the interquartile range above the third quartile or below the first quartile. Comparisons between the precision across sessions and recall across models were conducted using a two-tailed Wilcoxon signed-rank test with Bonferroni correction for multiple comparisons (*** if $p < 0.001$, * if $p < 0.05$). P-values: 0.0437 (A, Pinky, 2-class), 0.0108 (A, Thumb, 3-class), 0.0009 (B, Thumb, 2-class), 0.0004 (B, Pinky, 2-class), 0.0280 (B, Thumb, 3-class), 0.0270 (B, Index, 3-class), 2.09e-05 (B, Pinky, 3-class). (**B**) Group-level precision of 2-finger and 3-finger online ME decoding results between different classifiers at the group level. (**C** and **D**) Confusion matrixes for 2-finger (**C**) and 3-finger (**D**) online ME decoding results across sessions and different model types. From left to right are Session 1 with the base model, Session 1 with the fine-tuned model, Session 2 with the base model, and Session 2 with the fine-tuned model, respectively.

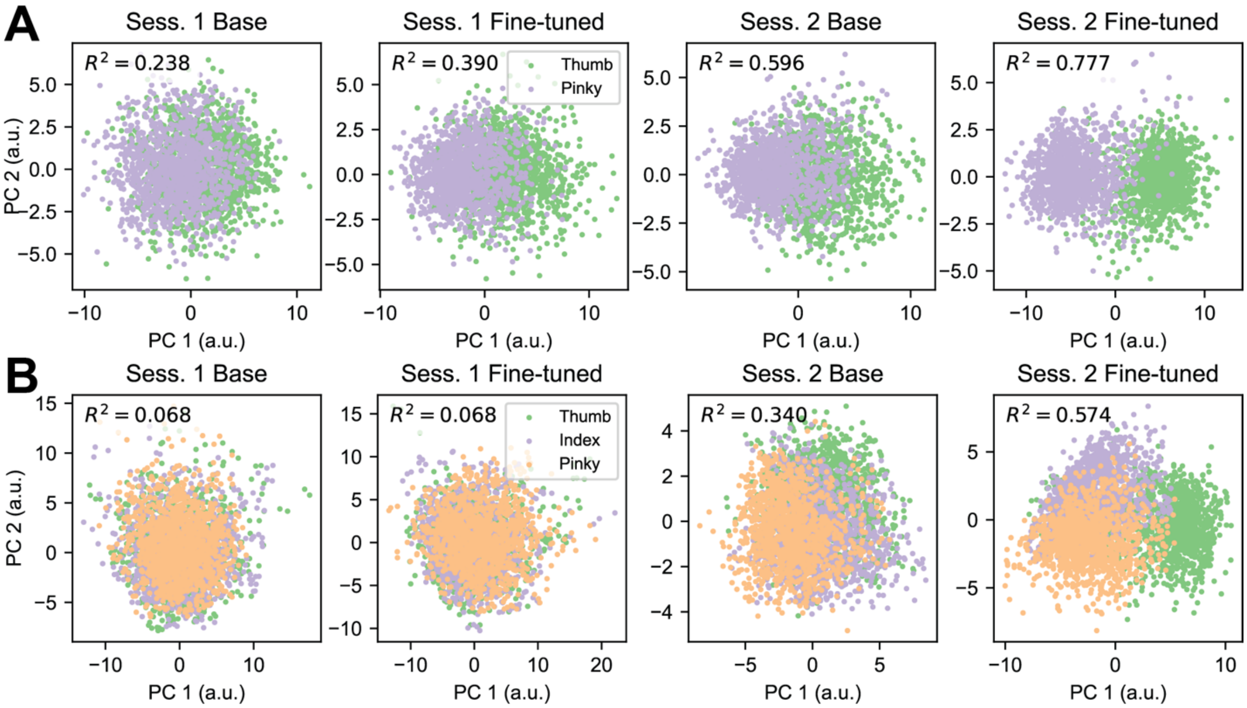

Supplementary Fig. S6. Example feature maps for deep learning online ME decoding. (A) Distribution of the first two principal components from PCA of intermediate-layer features extracted from the flatten layer outputs of the deep learning decoder for 2-class ME tasks. The R-squared score from linear regression between the intermediate-layer features and the task labels was annotated in the figure. **(B)** Distribution of the first two principal components from PCA of intermediate-layer features extracted from the flattened layer outputs for 3-class ME tasks. Class discriminability was enhanced through fine-tuning and the increased training size in both 2-class and 3-class scenarios.

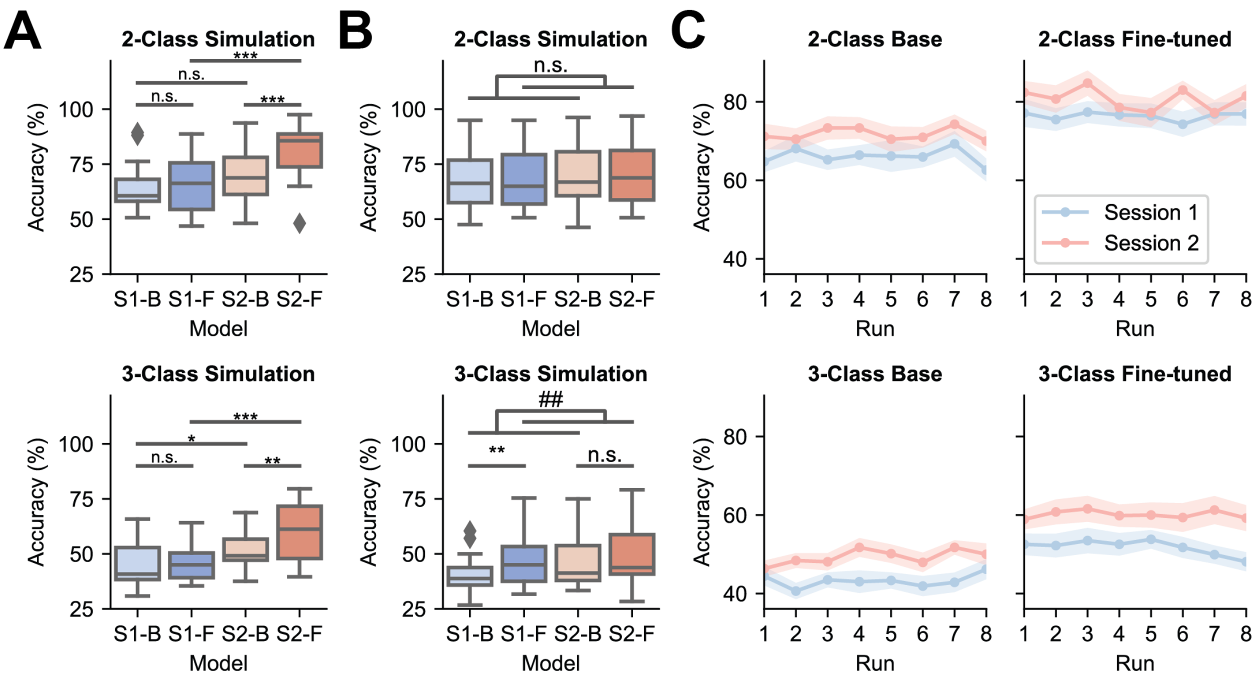

Supplementary Fig. S7. Machine and human learning effects for ME-based robotic control tasks. (**A**) Offline simulation results (n = 21 subjects) for Session 2 fine-tuned runs using Session 1 base models (S1-B), Session 1 fine-tuned models (S1-F), and Session 2 base models (S2-B), as well as the online decoding results for Session 2 fine-tuned runs (S2-F). The center lines indicate the median value. The boxes extend from the lower quartile to the upper quartile. Diamonds indicate outliers that are more than 1.5 times the interquartile range above the third quartile or below the first quartile. Comparisons between the sessions and the models were made using a two-tailed Wilcoxon signed-rank test with Bonferroni multi-comparison correction (*** if $p < 0.001$, ** if $p < 0.01$, * if $p < 0.05$, n.s. if no statistical significance is found). P-values: 0.0004 (S2-B vs. S2-F, 2-class), 0.0006 (S1-F vs. S2-F, 2-class), 0.0038 (S2-B vs. S2-F, 3-class), 0.0147 (S1-B vs. S2-B, 3-class), 0.0002 (S1-F vs. S2-F, 3-class). (**B**) Offline FBCSP decoding results on ME robotic finger control data (n = 21 subjects). S1-B, S1-F, S2-B, and S2-F denote decoding results for Session 1 base runs, Session 1 fine-tuned runs, Session 2 base runs, and Session 2 fine-tuned runs respectively. The center lines indicate the median value. The boxes extend from the lower quartile to the upper quartile. Diamonds indicate outliers that are more than 1.5 times the interquartile range above the third quartile or below the first quartile. Statistical analysis using a two-way repeated-measures ANOVA (n = 21) with main effects of session and model. Main effect of the model: ### if $p < 0.001$, n.s. if no statistical significance is found. Post hoc comparisons between models within the same session were made using a two-tailed Wilcoxon signed-rank test with Bonferroni correction (*** if $p < 0.001$, ** if $p < 0.01$, * if $p < 0.05$, n.s. if no statistical significance is found). P-values: 0.0013 (model, 3-class), 0.0071 (S1, 3-class). (**C**) Group-averaged performance trends across eight consecutive online runs within the same paradigm. The center lines indicate the mean values. The shaded areas represent the standard error.

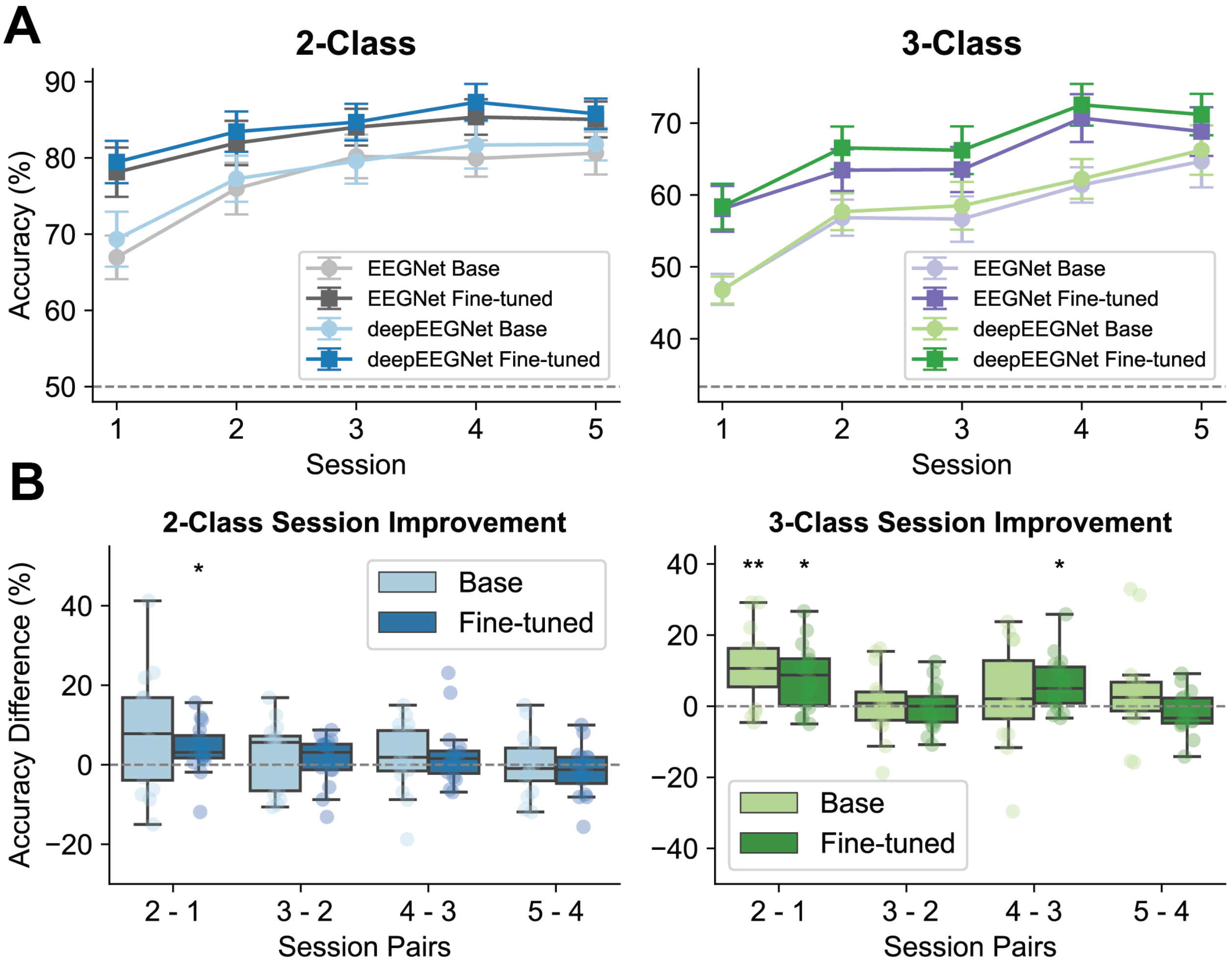

Supplementary Fig. S8. Simulated online decoding using deepEEGNet for MI-based robotic control tasks. (**A**) Simulated decoding accuracy using deepEEGNet for 2-finger and 3-finger MI over five online sessions, overlaid with the simulated decoding accuracy of EEGNet for comparison (n = 16 subjects). The grey dashed line represents the chance level, and error bars indicate the standard error. (**B**) Pairwise comparisons of deepEEGNet decoding accuracy between consecutive sessions for 2-finger and 3-finger MI (n = 16 subjects). The center lines indicate the median value. The boxes extend from the lower quartile to the upper quartile. The whiskers span up to 1.5 times the interquartile range. Performance improvements across sessions using deepEEGNet were statistically assessed with one-sided Wilcoxon signed-rank tests, conducted separately for each session pair and model type. Statistical significance after Bonferroni correction is denoted as ***$p < 0.001$, **$p < 0.01$, *$p < 0.05$. P-values: 0.0208 (2-class, 2-1, Fine-tuned), 0.0054 (3-class, 2-1, Base), 0.0122 (3-class, 4-3, Base), 0.0103 (3-class, 2-1, Fine-tuned).

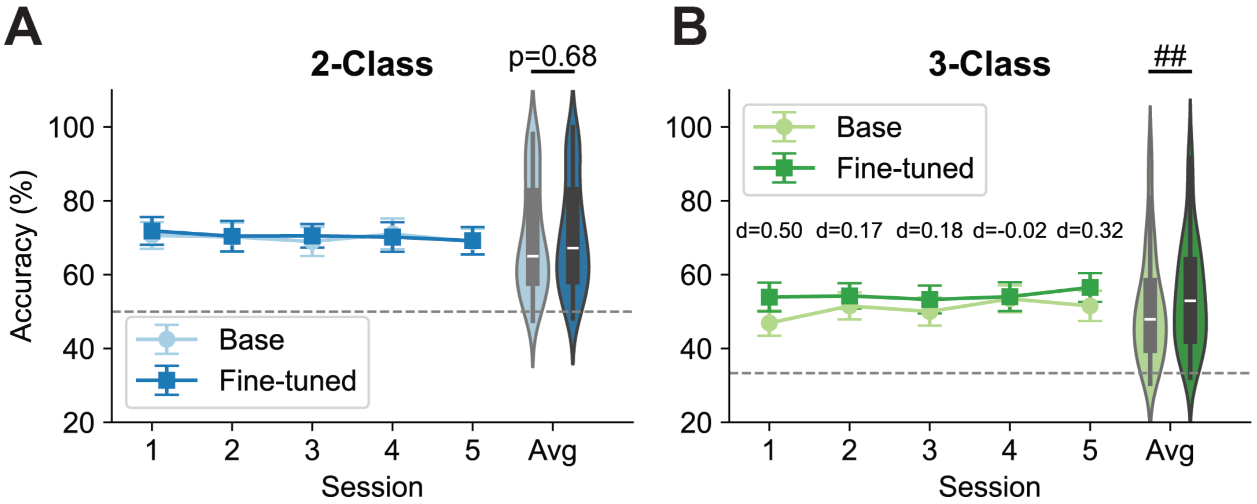

Supplementary Fig. S9. Offline FBCSP decoding results on MI robotic finger control data over 5 training sessions. (A) Group-level trends in 2-finger MI accuracy over five online sessions (n = 16 subjects). The grey dashed line represents the chance level, and the error bars indicate the standard error. The mean performance for the Base and Fine-tuned runs across all sessions is shown on the right. Statistical analysis using a two-way repeated-measures ANOVA with main effects of session and model. Main effect of the model: ## if p < 0.01. **(B)** Group-level trends in 3-finger MI accuracy over five online sessions (n = 16 subjects), with the mean performance for the Base and Fine-tuned runs displayed on the right. Cohen's *d* for the decoding accuracy distribution between the Base and Fine-tuned runs for each session was annotated above the line plot. P-value: 0.0016.

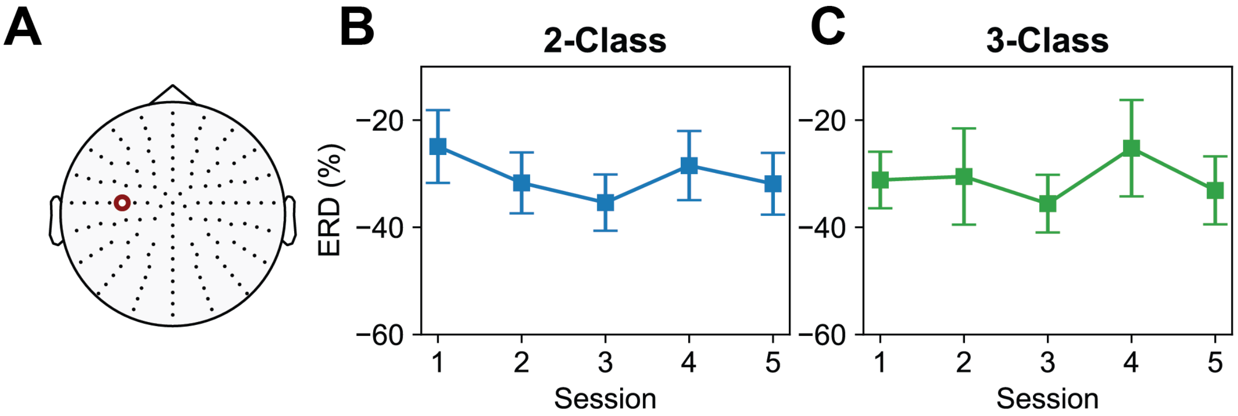

Supplementary Fig. S10. Alpha ERD at channel C3 during MI robotic finger control. (**A**) EEG electrode layout with the position of channel C3, deemed to elicit the strongest contralateral ERD during right-hand MI tasks, marked in dark red. (**B**) Group-level alpha band ERD (8 - 13 Hz) for 2-finger MI tasks across 5 online sessions (n = 16 subjects). Error bars represent the standard error. One-way ANOVA indicated non-significant session effects (F = 0.432, p = 0.784). (**C**) Group-level alpha band ERD (8 - 13 Hz) for 3-finger MI tasks across 5 online sessions (n = 16 subjects). One-way ANOVA indicated non-significant session effects (F = 0.284, p = 0.887).

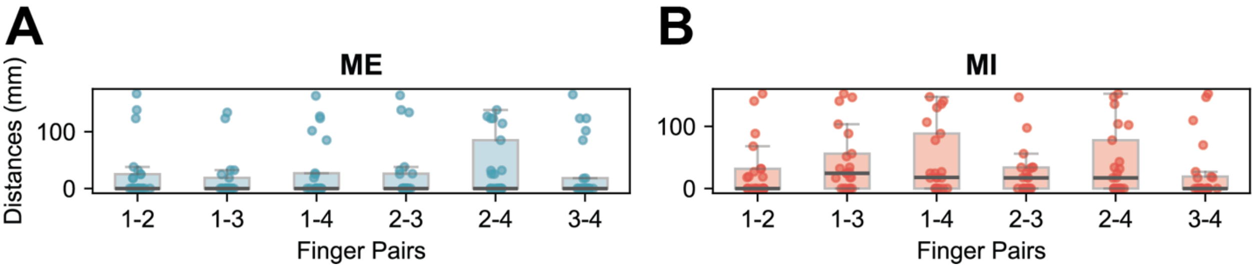

Supplementary Fig. S11. Inter-finger distance for ERD activation. (**A**) Distribution of the distance between EEG channels with the maximum alpha ERD activation during offline finger ME tasks (n = 21 subjects). The center lines indicate the median value. The boxes extend from the lower quartile to the upper quartile. The whiskers span up to 1.5 times the interquartile range. (**B**) Distribution of the distance between EEG channels with the maximum alpha ERD activation during offline finger MI tasks (n = 21 subjects).

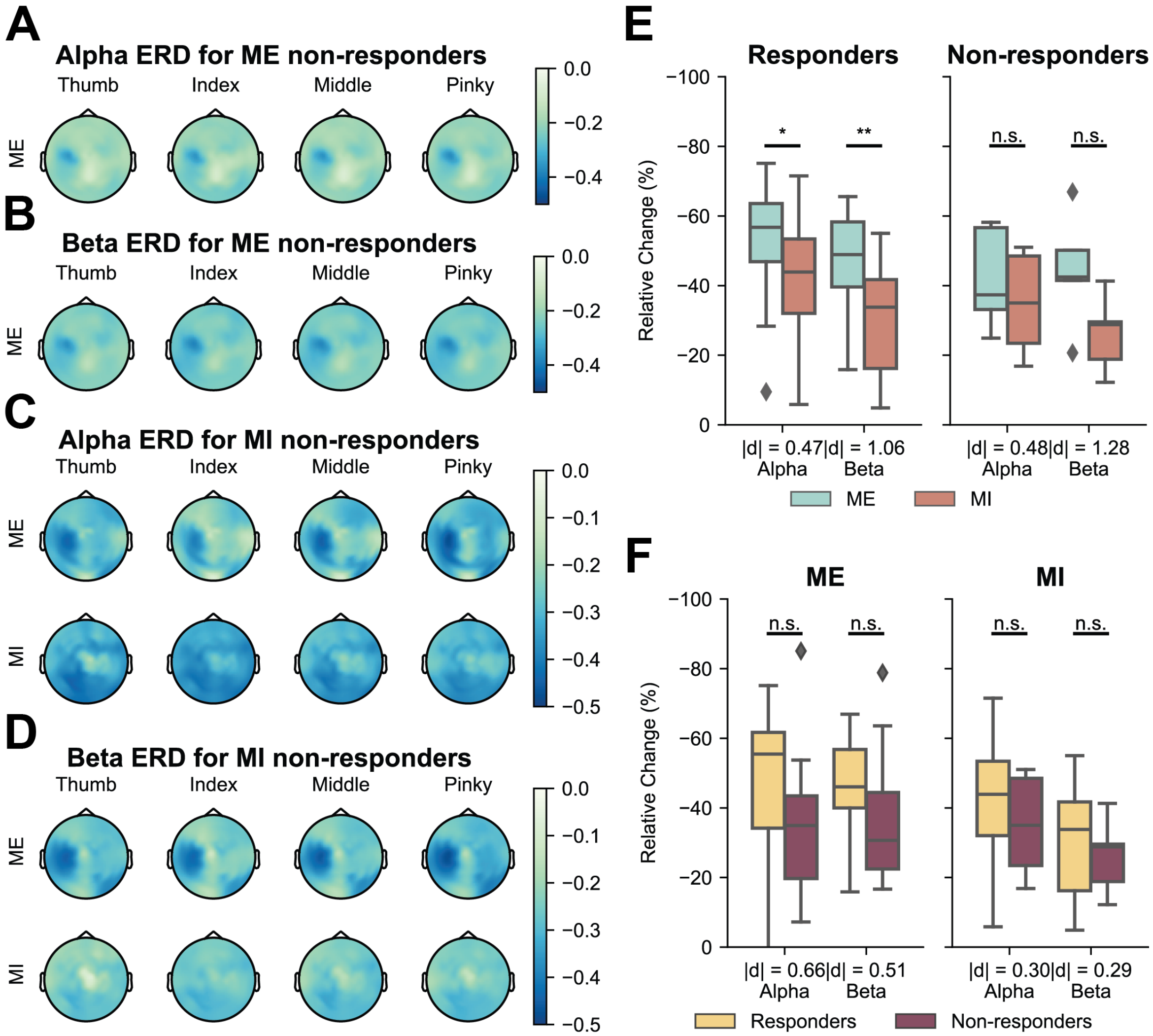

Supplementary Fig. S12. Comparison of electrophysiological activations between BCI responders and non-responders. (**A** and **B**) Group-level alpha band (8 - 13 Hz) (**A**) and beta band (13 - 30 Hz) (**B**) event-related desynchronization (ERD) topographies during finger ME tasks for ME non-responders (n = 13 subjects). From left to right, ERD topographies corresponding to thumb, index, middle, and pinky movements are displayed. (**C** and **D**) Group-level task-specific alpha (**C**) and beta band (**D**) ERD topographies for MI non-responders (n = 5 subjects). The top row presents results from ME data, while the bottom row presents results from MI data. (**E**) Comparison of the ERD magnitude between ME and MI conditions for both BCI responders and non-responders. The center lines indicate the median value. The boxes extend from the lower quartile to the upper quartile. Diamonds indicate outliers that are more than 1.5 times the interquartile range above the third quartile or below the first quartile. Statistical analysis was conducted using a two-tailed Wilcoxon signed-rank test with Bonferroni correction (* if $p < 0.05$, n.s. if no statistical significance is found). The effect size, Cohen's *d* for the ERD magnitude distribution between the ME and MI conditions, is indicated under each pair of bars. P-values: 0.0283 (Alpha), 0.0097 (Beta). (**F**) Comparison of the ERD magnitude between BCI responders and non-responders for both ME and MI conditions. Statistical analysis was conducted using a two-sided T-test for the means of two independent samples of scores with Bonferroni correction (n.s. if no statistical significance is found). The effect size, Cohen's *d* for the ERD magnitude distribution between responders and non-responders, is indicated under each pair of bars.

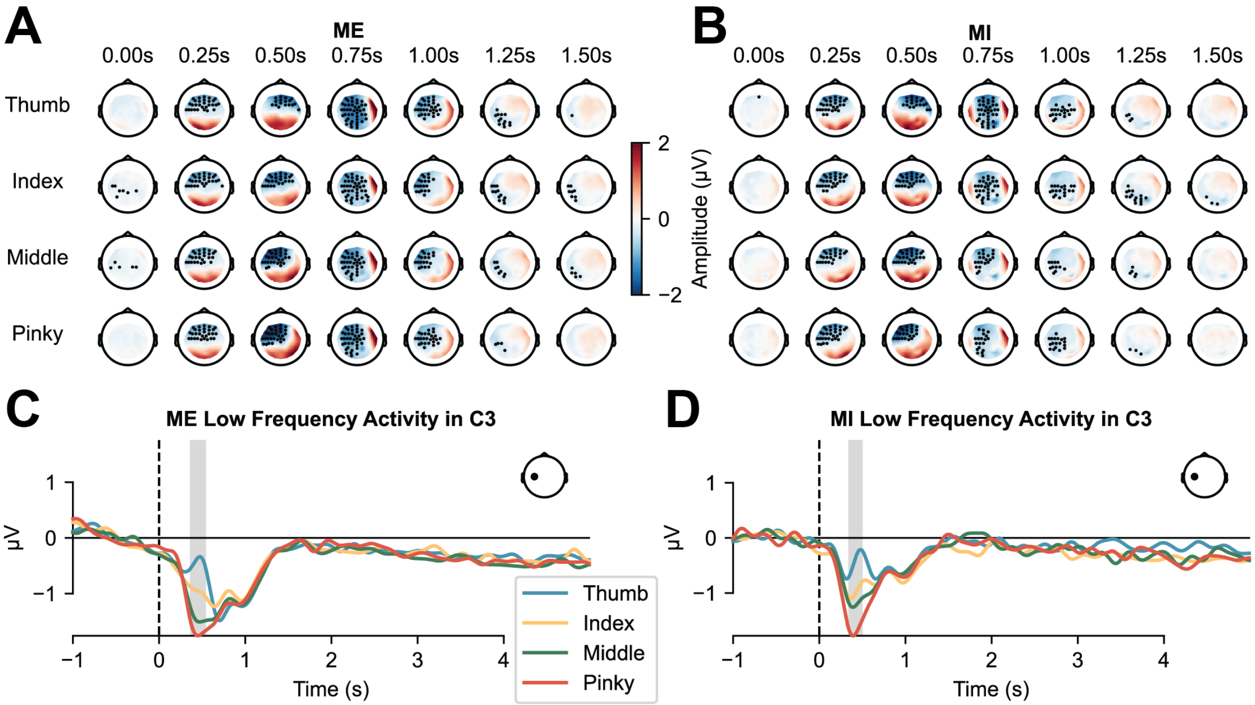

Supplementary Fig. S13. Low-frequency EEG activity during finger ME and MI tasks. (**A** and **B**) Group-averaged topographical maps of low-frequency (0.3–3 Hz) EEG amplitude within the first 1.5 seconds following trial onset for finger ME (**A**) and MI (**B**). Channels showing statistically significant differences from baseline amplitude, identified via cluster-level permutation testing, are marked in black. (**C** and **D**) MRCPs at channel C3 for different finger movements during ME (**C**) and MI (**D**). The shaded area highlights a significant amplitude difference among the four fingers ($p < 0.05$, uncorrected for multiple comparisons), based on a one-way repeated-measures ANOVA.

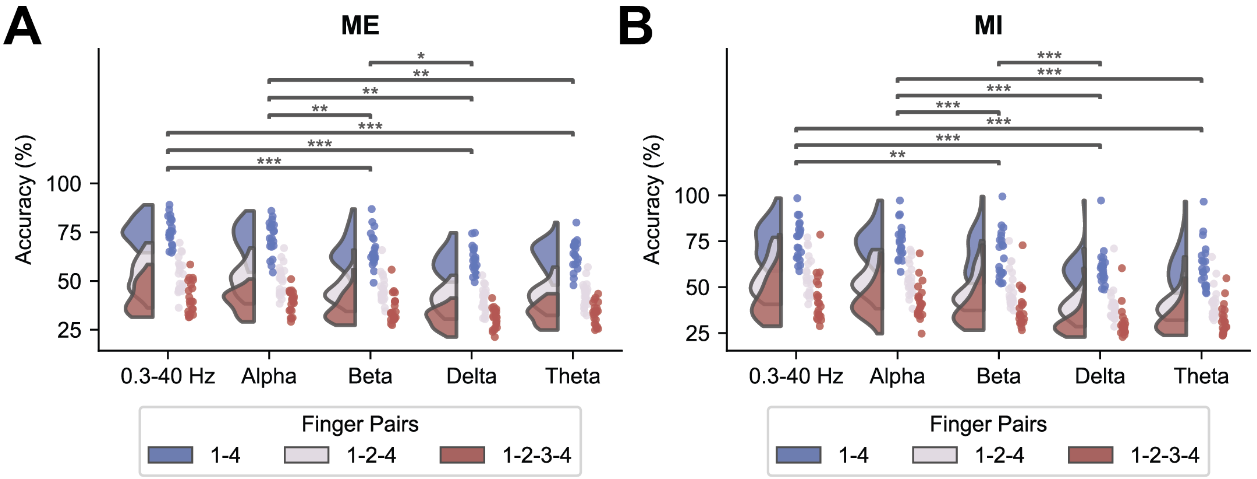

Supplementary Fig. S14. Comparison of offline EEGNet decoding performance for ME (A) and MI (B) tasks using EEG signals filtered with different bandpass settings (n = 21 subjects). The x-axis shows classification results for different frequency bands (0.3 – 40 Hz, alpha band, beta band, delta band, theta band). Offline classifications were performed on thumb vs. pinky (1-4), thumb vs. index finger vs. pinky (1-2-4), and all four fingers (1-2-3-4). Two-way ANOVA was conducted across different frequency bands and finger pairs, and statistical significance was observed in all task conditions. Significance stars indicate post hoc pairwise comparison results using an FDR-corrected two-tailed Wilcoxon signed-rank test (*** if p<0.001, ** if p<0.01, * if p < 0.05). P-values: 4.76e-06 (A, 0.3-40 Hz vs. Beta), 4.76e-06 (A, 0.3-40 Hz vs. Delta), 1.58e-05 (A, 0.3-40 Hz vs. Theta), 0.0018 (A, Alpha vs. Beta), 0.0020 (A, Alpha vs. Delta), 0.0023 (A, Alpha vs. Theta), 0.0225 (A, Beta vs. Delta), 0.0046 (B, 0.3-40 Hz vs. Beta), 0.0001 (B, 0.3-40 Hz vs. Delta), 4.76e-06 (B, 0.3-40 Hz vs. Theta), 0.0003 (B, Alpha vs. Beta), 4.76e-06 (B, Alpha vs. Delta), 7.94e-05 (B, Alpha vs. Theta), 0.0001 (B, Beta vs. Delta).

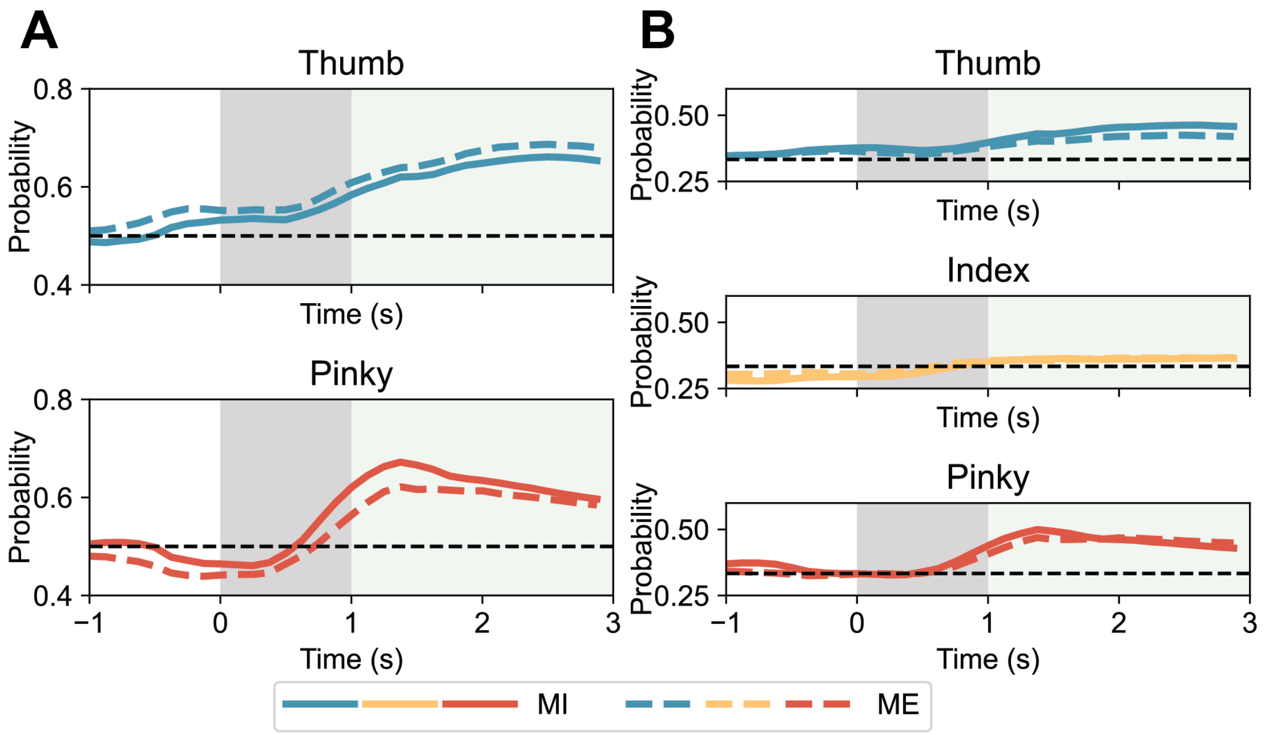

Supplementary Fig. S15. Group averaged decoding probability of the target class over time. (**A**) The ME and MI online 2-class decoding probability of the target classes at the group level (n = 21 subjects). The black dashed line indicates the chance level. The grey shaded area represents the first second after the trial onset before the feedback begins; the green shaded area represents the feedback period. (**B**) The ME and MI online 3-class decoding probability of the target classes at the group level (n = 21 subjects).

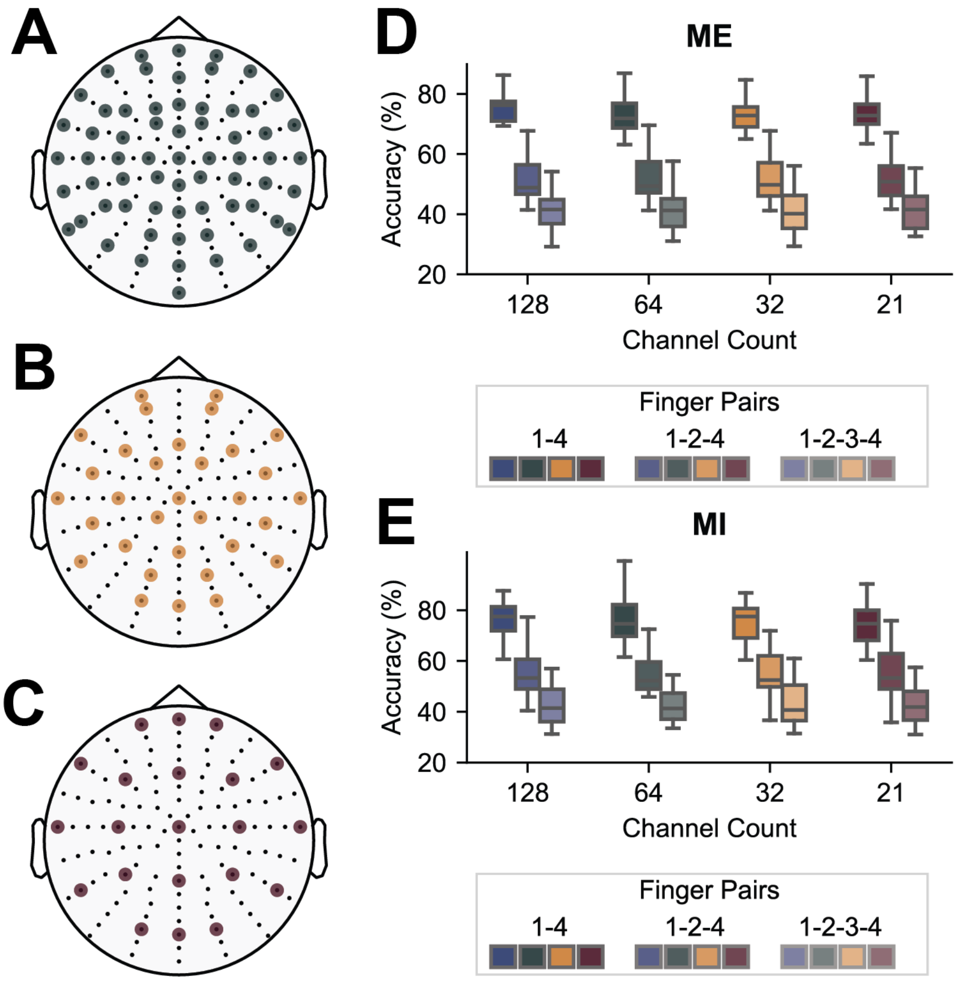

Supplementary Fig. S16. The effect of EEG input with different channel densities on offline Finger ME and MI decoding performance. (**A**, **B**, and **C**) 128-channel EEG electrode layout, with 64 channels (**A**), 32 channels (**B**), and 21 channels (**C**) following the 10-20 international electrode layout overlaid in dark green, yellow, and dark red. (**D** and **E**) Offline decoding results for finger ME (**D**) and MI (**E**) tasks (n = 21 subjects). Decoding performance was compared using EEG inputs with different channel counts, including 128 channels, in which all the recorded electrodes were used, 64 channels, 32 channels, and 21 channels, following the layouts in (A, B, and C). The center lines indicate the median value. The boxes extend from the lower quartile to the upper quartile. The whiskers span up to 1.5 times the interquartile range. A two-way repeated-measures ANOVA found no significant main effect of Channel Count for ME ($F = 1.653$, $p = 0.186$) and MI ($F = 0.296$, $p = 0.827$) tasks.

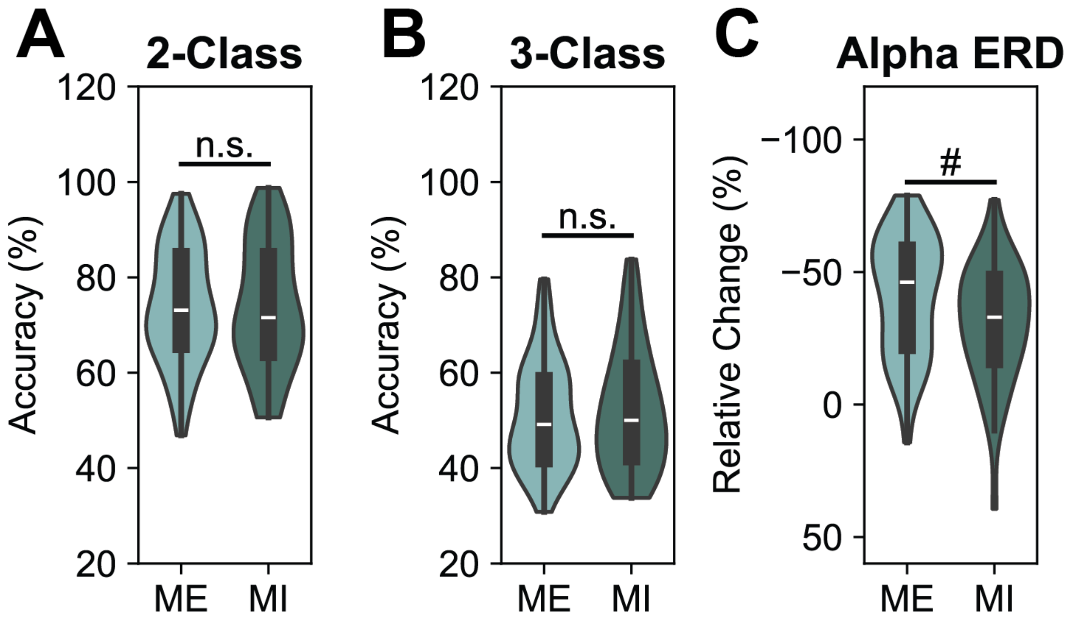

Supplementary Fig. S17. Comparison of online performance between ME and MI. (**A**) Comparison between 2-finger ME online performance and 2-finger MI online performance (n = 21 subjects). The center lines indicate the median value. The boxes extend from the lower quartile to the upper quartile. The whiskers span up to 1.5 times the interquartile range. Statistical analysis using a two-way repeated-measures ANOVA with main effects of session and task (ME vs. MI). Main effect of the task: n.s. no statistical significance found. (**B**) Comparison between 3-finger ME online performance and 3-finger MI online performance (n = 21 subjects). Main effect of the task: n.s. no statistical significance found.(**C**) Comparison of alpha band ERD (8 - 13 Hz) at channel C3 for online ME task and online MI task (n = 21 subjects). Main effect of the task: # if $p < 0.05$. P-value: 0.0382.

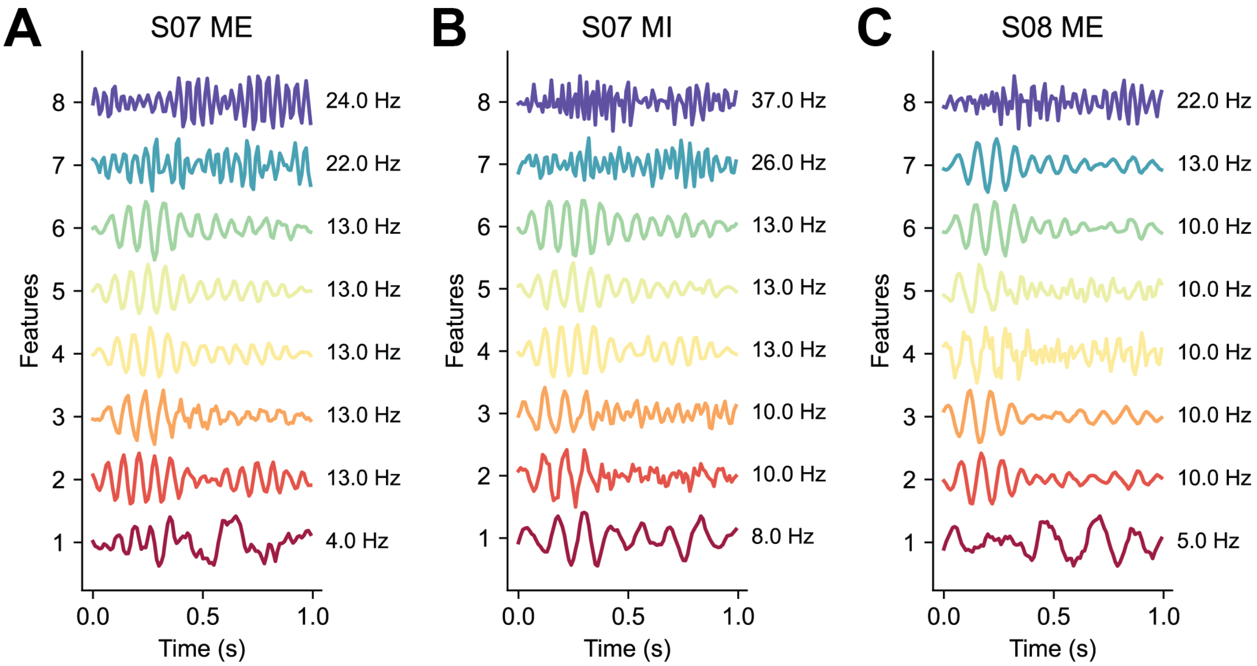

Supplementary Fig. S18. Visualization of the temporal features derived from within-subject trained EEGNet-8,2 models for Subject 07 during the finger ME task (A), Subject 07 during the finger MI task (B), and Subject 08 during the finger ME task (C). Each row represents a learned temporal kernel from the temporal convolution layer, derived from a 1-second input window. Kernels are sorted by their dominant frequency, which is indicated to the right of each corresponding feature.

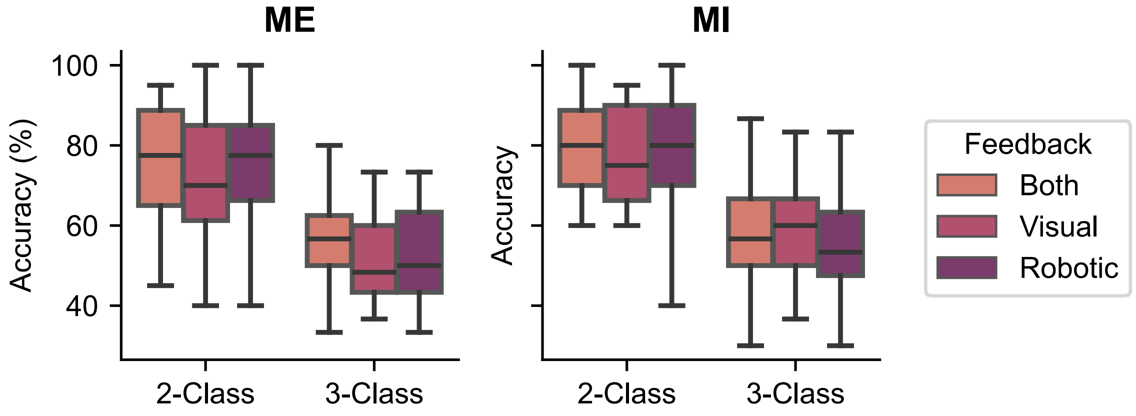

Supplementary Fig. S19. Online performance comparison under different feedback conditions (n = 10 subjects). Group-level online performance for 2-finger ME, 3-finger ME, 2-finger MI, and 3-finger MI tasks comparing three feedback conditions. “Both” indicates a combination of the visual feedback on the screen and the robotic feedback; “Visual” refers to visual feedback only; “Robotic” indicates robotic feedback only. The center lines indicate the median value. The boxes extend from the lower quartile to the upper quartile. The whiskers span up to 1.5 times the interquartile range. Statistical analysis was performed using a two-way repeated-measures ANOVA with the main effects of feedback and task (2-Class vs. 3-Class) on ME and MI data separately. No statistical significance was found on the main effect of the feedback (ME: $F = 0.592$, $p = 0.556$; MI: $F = 0.105$, $p = 0.900$).

Supplementary Table S1. Model architecture for deepEEGNet.

Layer	# filters	Kernel size
Input		
Conv2D	16	(1, 64)
BatchNorm		
DepthwiseConv2D	32	(128, 1)
BatchNorm		
Activation (ELU)		
AveragePooling2D		(1, 4)
Dropout (p = 0.65)		
SeparableConv2D	32	(1, 16)
BatchNorm		
Activation (ELU)		
AveragePooling2D		(1, 2)
Dropout (p = 0.65)		
SeparableConv2D	64	(1, 8)
BatchNorm		
Activation (ELU)		
AveragePooling2D		(1, 2)
Dropout (p = 0.65)		
SeparableConv2D	32	(1, 16)
BatchNorm		
Activation (ELU)		
AveragePooling2D		(1, 2)
Dropout (p = 0.65)		
Flatten		
Dense		
Activation (softmax)		
