# Supplementary material for: EEG-based brain-computer interface enables real-time robotic hand control at individual finger level
Source: Nat Commun. 2025 Jun 30;16:5401. doi: 10.1038/s41467-025-61064-x (PMC12209421; doi:10.1038/s41467-025-61064-x)
Supplement: Supplementary file 2 — Description of Additional Supplementary Files [file 41467_2025_61064_MOESM2_ESM.pdf]

Description of Additional Supplementary Files

Supplementary Movie 1. Online example of continuous MI-based 2-class robotic finger control (thumb vs. pinky) without online smoothing.

Supplementary Movie 2. Online example of continuous MI-based 3-class robotic finger control (thumb, index, and pinky) without online smoothing.

Supplementary Movie 3. Online example of continuous ME-based 2-class robotic finger control (thumb vs. pinky) with online smoothing.

Supplementary Movie 4. Online example of continuous ME-based 3-class robotic finger control (thumb, index, and pinky) with online smoothing
