# Supplementary material for: EEG-based brain-computer interface enables real-time robotic hand control at individual finger level
Source: Nat Commun. 2025 Jun 30;16:5401. doi: 10.1038/s41467-025-61064-x (PMC12209421; doi:10.1038/s41467-025-61064-x)
Supplement: Supplementary file 7 — Reporting Summary [file 41467_2025_61064_MOESM7_ESM.pdf]

nature portfolio

Corresponding author(s): Bin He

Last updated by author(s): May 21, 2025

Reporting Summary

Nature Portfolio wishes to improve the reproducibility of the work that we publish. This form provides structure for consistency and transparency in reporting. For further information on Nature Portfolio policies, see our Editorial Policies and the Editorial Policy Checklist.

Statistics

For all statistical analyses, confirm that the following items are present in the figure legend, table legend, main text, or Methods section.

n/a	Confirmed	
☐	☒	The exact sample size (*n*) for each experimental group/condition, given as a discrete number and unit of measurement
☐	☒	A statement on whether measurements were taken from distinct samples or whether the same sample was measured repeatedly
☐	☒	The statistical test(s) used AND whether they are one- or two-sided<br>*Only common tests should be described solely by name; describe more complex techniques in the Methods section.*
☐	☒	A description of all covariates tested
☐	☒	A description of any assumptions or corrections, such as tests of normality and adjustment for multiple comparisons
☐	☒	A full description of the statistical parameters including central tendency (e.g. means) or other basic estimates (e.g. regression coefficient) AND variation (e.g. standard deviation) or associated estimates of uncertainty (e.g. confidence intervals)
☐	☒	For null hypothesis testing, the test statistic (e.g. *F*, *t*, *r*) with confidence intervals, effect sizes, degrees of freedom and *P* value noted<br>*Give P values as exact values whenever suitable.*
☒	☐	For Bayesian analysis, information on the choice of priors and Markov chain Monte Carlo settings
☐	☒	For hierarchical and complex designs, identification of the appropriate level for tests and full reporting of outcomes
☐	☒	Estimates of effect sizes (e.g. Cohen's *d*, Pearson's *r*), indicating how they were calculated

Our web collection on statistics for biologists contains articles on many of the points above.

Software and code

Policy information about availability of computer code

Data collection	EEG data were acquired using BCI2000.
Data analysis	Data analysis was conducted using custom scripts developed in Python (version 3.10.13) and MATLAB (R2023a). Online processing and classification were performed using custom Python (version 3.8.4) scripts made for BCPy2000 (2021.1.0), a part of the BCI2000 program.

For manuscripts utilizing custom algorithms or software that are central to the research but not yet described in published literature, software must be made available to editors and reviewers. We strongly encourage code deposition in a community repository (e.g. GitHub). See the Nature Portfolio guidelines for submitting code & software for further information.

Data

Policy information about availability of data

All manuscripts must include a data availability statement. This statement should provide the following information, where applicable:

- Accession codes, unique identifiers, or web links for publicly available datasets
- A description of any restrictions on data availability
- For clinical datasets or third party data, please ensure that the statement adheres to our policy

Data supporting the conclusions are included in the paper and Supplementary Materials. Additional EEG data in all subjects will be available in Figshare when the paper is accepted.

Research involving human participants, their data, or biological material

Policy information about studies with human participants or human data. See also policy information about sex, gender (identity/presentation), and sexual orientation and race, ethnicity and racism.

Reporting on sex and gender	Self-reported sex data were collected from participants who consented to provide this information. However, sex was not considered a primary factor in the study design or analysis, and no sex-based analyses were performed. The study focused on able-bodied volunteers participating in BCI experiments, and outcomes were not expected to differ by sex.
Reporting on race, ethnicity, or other socially relevant groupings	Self-reported race data were collected from participants who consented to provide this information. These variables were not included in the study design or analysis. No race- or ethnicity-based analyses were conducted, and these variables were not used as proxies for other factors.
Population characteristics	Twenty-one right-handed able-bodied subjects were studied (six male / fifteen female; mean age: 24.23 ± 3.72)
Recruitment	Participants were recruited through the distribution of flyers across the university campus. All procedures and protocols were approved by the Institutional Review Board of Carnegie Mellon University (protocol number: STUDY2017_00000548). Before participating in the experiment, subjects completed screening forms to determine their eligibility and were informed of the potential risks associated with the study. Written informed consent was obtained from all subjects prior to the commencement of the experiment.
Ethics oversight	Carnegie Mellon University

Note that full information on the approval of the study protocol must also be provided in the manuscript.

Field-specific reporting

Please select the one below that is the best fit for your research. If you are not sure, read the appropriate sections before making your selection.

☐ Life sciences ☒ Behavioural & social sciences ☐ Ecological, evolutionary & environmental sciences

For a reference copy of the document with all sections, see nature.com/documents/nr-reporting-summary-flat.pdf

Behavioural & social sciences study design

All studies must disclose on these points even when the disclosure is negative.

Study description	Quantitative experimental
Research sample	Twenty-one right-handed able-bodied subjects were studied (six male / fifteen female; mean age: 24.23 ± 3.72). Eligibility was restricted to right-handed individuals to align with the focus on right-hand motor execution and imagery. This criterion was implemented to control for potential confounding effects of handedness on task performance.
Sampling strategy	Participants were recruited using a convenience sampling approach from the local population of able-bodied adults. No formal sample size calculation was performed. The chosen sample size was determined empirically based on prior studies in noninvasive BCI research, which demonstrated that similar sample sizes were sufficient to observe robust neural decoding effects. The sample size was deemed adequate to achieve the aims of the study and to ensure meaningful statistical analysis.
Data collection	EEG data were recorded using a 128-channel BioSemi EEG headcap and an ActiveTwo amplifier (BioSemi, Amsterdam, The Netherlands), with data acquisition performed on a personal computer. With participant consent, a video camera was also used to record experimental sessions. Data collection took place in a private experimental room with only the participant and the researcher present. The researcher was aware of the experimental conditions and study hypothesis during data collection.
Timing	The data collection started on 11/26/2023 and ended on 4/3/2025.
Data exclusions	Eighteen subjects were excluded from the entire study due to unsatisfactory performance as a BCI responder (<70% decoding accuracy in binary classifications) in either the finger ME or MI offline sessions. The exclusion criteria was pre-established. This exclusion process was designed to address the issue that there can be ~30% subjects are non-responders to sensorimotor rhythm BCI, and our goal is to study if and how an individual can control a robotic finger among BCI responders.
Non-participation	Ten subjects withdrew from the study due to scheduling conflicts.
Randomization	Participants were not allocated into experimental groups.

Reporting for specific materials, systems and methods

We require information from authors about some types of materials, experimental systems and methods used in many studies. Here, indicate whether each material, system or method listed is relevant to your study. If you are not sure if a list item applies to your research, read the appropriate section before selecting a response.

Materials & experimental systems

n/a	Involved in the study
☒	☐ Antibodies
☒	☐ Eukaryotic cell lines
☒	☐ Palaeontology and archaeology
☒	☐ Animals and other organisms
☒	☐ Clinical data
☒	☐ Dual use research of concern
☒	☐ Plants

Methods

n/a	Involved in the study
☒	☐ ChIP-seq
☒	☐ Flow cytometry
☒	☐ MRI-based neuroimaging

Plants

Seed stocks	n/a
Novel plant genotypes	n/a
Authentication	n/a
